# *qEMF3*, a novel QTL for the early-morning flowering trait from wild rice, *Oryza officinalis*, to mitigate heat stress damage at flowering in rice, *O. sativa*

**DOI:** 10.1093/jxb/eru474

**Published:** 2014-12-22

**Authors:** Hideyuki Hirabayashi, Kazuhiro Sasaki, Takashi Kambe, Ritchel B. Gannaban, Monaliza A. Miras, Merlyn S. Mendioro, Eliza V. Simon, Patrick D. Lumanglas, Daisuke Fujita, Yoko Takemoto-Kuno, Yoshinobu Takeuchi, Ryota Kaji, Motohiko Kondo, Nobuya Kobayashi, Tsugufumi Ogawa, Ikuo Ando, Krishna S. V. Jagadish, Tsutomu Ishimaru

**Affiliations:** ^1^NARO Institute of Crop Science, NARO, 2-1-18 Kannondai, Tsukuba, Ibaraki 305-8518, Japan; ^2^Japan International Research Centre for Agricultural Sciences (JIRCAS), 1-1 Ohwashi, Tsukuba, Ibaraki 305-8686, Japan; ^3^International Rice Research Institute (IRRI), DAPO Box 7777, Metro Manila, The Philippines; ^4^The University of Tokyo, Graduate School of Agricultural and Life Sciences, Institute of Sustainable Agro-ecosystem Services (ISAS), 1-1-1 Midoricho, Nishitokyo, Tokyo 188-0002, Japan; ^5^University of the Philippines, Los Baños, Laguna, The Philippines; ^6^NARO Tohoku Agricultural Research Centre (TARC), NARO, Shimo Furumichi, Daisen, Akita 014-0102, Japan; ^7^Faculty of Agriculture, University of Miyazaki, 1-1 Gakuen kihanadai nishi, Miyazaki, Miyazaki 889-2192, Japan

**Keywords:** Early-morning flowering (EMF), flower opening time (FOT), global warming, heat stress, quantitative trait locus (QTL), rice (*Oryza sativa* L.), spikelet sterility.

## Abstract

A decline in rice (*Oryza sativa* L.) production caused by heat stress is one of the biggest concerns resulting from future climate change. Rice spikelets are most susceptible to heat stress at flowering. The early-morning flowering (EMF) trait mitigates heat-induced spikelet sterility at the flowering stage by escaping heat stress during the daytime. We attempted to develop near-isogenic lines (NILs) for EMF in the *indica*-type genetic background by exploiting the EMF locus from wild rice, *O. officinalis* (CC genome). A stable quantitative trait locus (QTL) for flower opening time (FOT) was detected on chromosome 3. A QTL was designated as *qEMF3* and it shifted FOT by 1.5–2.0h earlier for cv. Nanjing 11 in temperate Japan and cv. IR64 in the Philippine tropics. NILs for EMF mitigated heat-induced spikelet sterility under elevated temperature conditions completing flower opening before reaching 35°C, a general threshold value leading to spikelet sterility. Quantification of FOT of cultivars popular in the tropics and subtropics did not reveal the EMF trait in any of the cultivars tested, suggesting that *qEMF3* has the potential to advance FOT of currently popular cultivars to escape heat stress at flowering under future hotter climates. This is the first report to examine rice with the EMF trait through marker-assisted breeding using wild rice as a genetic resource.

## Highlight

*qEMF3*, a novel QTL for the early-morning flowering trait to mitigate heat-induced spikelet sterility at flowering in rice, was identified using a wild rice, *Oryza officinalis*, as a genetic resource.

## Introduction

Rice (*Oryza sativa* L.) is one of the most important staple food crops for nearly half the world’s population (Carriger and Vallee, 2007). Predicted future climate change is expected to have a negative impact on global rice production. Maximum and minimum daily temperatures, and the number of hot days and warm nights in a year, are estimated to increase over most land areas (IPCC, 2013). In addition, climate variability is predicted to increase, leading to frequent episodes of heat stress, often coinciding with key developmental stages in crops, such as flowering. Seasonal temperatures in the tropics and subtropics by the end of the 21st century are predicted to exceed the most extreme seasonal temperatures recorded in the past (Battisti and Naylor, 2009). Breeding crops able to cope with future climate change is a pressing requirement to feed the growing population in the era of global warming.

High temperatures (>35°C) at flowering cause spikelet sterility in rice (Satake and Yoshida, 1978; Matsui *et al.*, 1997). Heat stress for 1h induces spikelet sterility if it coincides with flowering (Jagadish *et al.*, 2007). Exposure of flowering spikelets to heat stress results in the failure of anther dehiscence and the reduced number of germinating pollen grains on the stigma (Satake and Yoshida 1978; Matsui *et al.*, 2001*a*; Matsui and Omasa, 2002; Jagadish *et al.*, 2010). Spikelet sterility positively correlates with a yield reduction in experimental paddy fields (Kim *et al.*, 1996; Oh-e *et al.*, 2007), in line with the yield reduction caused by heat-induced spikelet sterility observed in tropical Asia (Osada *et al.*, 1973) and Africa (Matsushima *et al.*, 1982). In Bangladesh, eastern India, southern Myanmar, and northern Thailand, the flowering and early grain-filling stages of rice are predicted to coincide with high-temperature conditions, according to the local cropping patterns and crop calendars (Maclean *et al.*, 2002; Wassmann *et al.*, 2009). In the Jianghan Basin in China, significant losses in seed set were observed in local hybrid rice because of hot and humid conditions during flowering (Tian *et al.*, 2010). Damage to rice productivity because of heat stress at flowering has also been reported in temperate regions of Japan: in comparison with normal summers, spikelet sterility was significantly higher by 15–20% in the summer of 2007, when maximum temperatures reached a record level of 40°C during the flowering stage (Hasegawa *et al.*, 2011). Thus, heat stress at flowering poses a real threat to sustained rice production not only in tropical and subtropical regions, but also in temperate regions. Existing breeding programmes are challenged to develop rice that would be heat-resilient at flowering and could be grown across temperate, tropical, and subtropical regions.

To mitigate heat-induced spikelet sterility, two strategies have been proposed. One is to develop cultivars that shed larger numbers of pollen grains or produce pollen grains able to germinate at high temperatures. At the flowering stage, rice cultivars have different tolerance to high temperatures (Satake and Yoshida, 1978; Matsui *et al.*, 2001*b*; Prasad *et al.*, 2006; Jagadish *et al.*, 2008). A highly heat-tolerant cultivar, N22 (Satake and Yoshida, 1978; Mackill *et al.*, 1982; Jagadish *et al.*, 2010), has the tolerance allele *qHTSF4.1*, a QTL on chromosome 4, as a major genetic factor (Ye *et al.*, 2012). Another strategy, which is less explored, is to breed cultivars that escape heat at flowering because of their early-morning ﬂowering (EMF) trait (Satake and Yoshida, 1978). Spikelets are highly susceptible to heat stress at flowering; however, they remain fertile when flowering occurs 1h prior to heat stress, because fertilization is completed within 1h after the onset of flowering (Satake and Yoshida, 1978). The EMF strategy has been used to produce an introgression line, EMF20, with the EMF trait transferred from wild rice, *O. officinalis*, into the genetic background of *O. sativa* cv. Koshihikari (Ishimaru *et al.*, 2010). EMF20 flowered a few hours earlier than Koshihikari, which reduced heat-induced sterility at flowering at elevated temperatures in a greenhouse test (Ishimaru *et al.*, 2010).

Within the genus *Oryza*, the flower opening time (FOT) ranges widely from early morning to midnight. There are many accessions in wild rice with the EMF trait (Sheehy *et al.*, 2005). CG14 (*O. glaberrima*) has been reported as an EMF cultivar (Jagadish *et al.*, 2008). By using backcrossed inbred lines derived from a cross between *O. rufipogon* and *O. sativa*, QTLs for FOT have been detected on chromosomes 4, 5, and 10 (Thanh *et al.*, 2010). The *O. rufipogon* alleles of all QTLs theoretically contributed to the 30min advancement of FOT in *O. sativa* (Thanh *et al.*, 2010). However, the effect of the QTLs detected is yet to be proven to be materialized as a near-isogenic line (NIL) for EMF. Currently, there is no material available from ongoing rice breeding programmes that could be used to introduce a heat-escape trait to mitigate heat-induced spikelet sterility at flowering. In addition, there has been no systematic analysis of flowering dynamics of a large number of popular local cultivars occupying large areas in rice-growing regions, which would help to evaluate their heat-escape potential.

Hence, our work had three major objectives: (i) to identify QTLs for EMF and to develop NILs for EMF by using molecular markers and exploiting the EMF trait of the wild rice *O. officinalis*; (ii) to characterize the flowering pattern conferred by QTLs identified for EMF under various high-temperature regimes and at different geographical locations; and (iii) to quantify the FOT of popular cultivars grown across tropical and subtropical regions by systematic observation of their daily flowering patterns.

## Materials and methods

### Plant materials for QTL analysis

Rice plants were grown at the NARO Institute of Crop Science (NICS), Tsukuba (36° 02’N, 140° 10’E), Ibaraki, Japan. The introgression line, EMF20, was crossed with Nanjing 11 (Supplementary Figure S1A) because of the similar heading date in that location. The F_2_ population and self-pollinated F_3_ lines were derived from a cross between EMF20 and Nanjing 11 (Supplementary Figure S1A). The F_2_ seeds (in 2007) and F_3_ seeds (in 2008) were sown in seedling trays and 20-day-old seedlings were transplanted (146 F_2_ plants and 10 F_3_ plants into 250-cm^2^ pots, one plant per pot). As a basal dressing, a controlled-release fertilizer was applied in both experiments, which contained N (0.5g), P_2_O_5_ (2.3g), and K_2_O (2.2g) in each pot. Plants were kept free from pests and diseases by preventive application of chemicals.

### FOT of F_2_ and F_3_ plants

To reduce the phenological variation, a short-day treatment (light conditions for 9h) was imposed on Nanjing 11, EMF20, F_2_ plants, and F_3_ lines for 2 weeks before the panicle-initiation stage. The heading date among all the above entries was narrowed down to just 9 days (from 1 to 9 August 2007). In addition, solar radiation and air temperature are known to strongly affect the daily flowering pattern (Kobayashi *et al.*, 2010; Julia and Dingkuhn, 2012), and hence to reduce the variation induced by environmental factors, FOT was recorded only on sunny days. Opened spikelets were visually counted every 20min using two panicles per plant per day and in three plants each from EMF20, Nanjing 11, and 146 F_2_ populations. The beginning of FOT (BFOT, the time when the first spikelet opened on a given day) and the peak of FOT (PFOT, the time when the largest percentage of spikelets were open on a given day) were recorded for at least 2 days in 2007 for each individual F_2_ plant; BFOT of 10 F_3_ progenies of each F_2_ plant was recorded daily in 2008 for 4 days. EMF20 and Nanjing 11 were stagger-sown every 3 days to make their heading dates cover the entire period of heading dates of the F2 and F3 plants. The BFOT and PFOT data were used for QTL analysis.

### DNA extraction, map construction, and QTL analyses

Plant DNA was extracted from young leaves of the F_2_ plants and their parents using the CTAB method (Murray and Thompson, 1980). A genetic map was developed using 154 simple sequence repeat (SSR) markers (McCouch *et al.*, 2002). DNA amplification was performed for 35 cycles of 94°C (1min), 55°C (2min), and 72°C (3min), and a final extension at 72°C for 7min (PTC100, BioRad) using Taq DNA Polymerase (Thermo Fisher Scientific, Inc.). Amplified DNA products were electrophoresed in a 3.0% agarose gel in 0.5×TBE buffer. Analysis of linkage between SSR markers and linkage map construction for QTL analysis were performed with MAPMAKER/EXP 3.0 software (Lander *et al.*, 1987). QTL analysis was performed with composite interval mapping analysis in the WinQTL Cartographer 2.5 software (Wang *et al.*, 2007). The optimal log of odds (LOD) threshold values obtained with the permutation value set at 1000 by WinQTL Cartographer were used to determine the presence of a putative QTL, the percentages of variation explained by the QTL, and the additive effect.

### Segregation analysis and development of NILs for the EMF locus

To confirm the effect of the QTL detected (*qEMF3*), segregation analysis for BFOT and PFOT was performed by using the BC_3_F_2_ population. By using SSR markers in the *qEMF3* region (Fig. 2A), BC_3_F_2_ plants were classified into EMF20-homozygous, Nanjing 11-homozygous, and heterozygous. BFOT and PFOT were recorded from 10 plants of each classified group mentioned above and Nanjing 11 (as described in the section ‘FOT of F_2_ and F_3_ plants’) from late July to mid-August 2009 in Japan. To develop NILs for *qEMF3*, EMF20-homozygous plants for *qEMF3* were selected from BC_3_F_2_ plants by using the same SSR markers used in the segregation analysis. From these BC_3_F_2_ plants, a plant that was Nanjing 11-homozygous for other chromosome segments was selected and used as a NIL (Nanjing 11+*qEMF3*) for the following experiments. The heading date of BC_2_F_1_ plants with heterozygous *qEMF8* was 2 months later than that of the recurrent parent, Nanjing 11, resulting in a failure to proceed to the BC_3_F_1_ generation.

Nanjing 11 is well adapted to temperate rice-growing regions. To facilitate field testing of the EMF trait in heat-vulnerable regions in the tropics and subtropics, *qEMF3* was transferred to IR64, a popular *indica*-type cultivar adapted for tropical and subtropical regions (Table 3; Khush, 1987). To develop IR64+*qEMF3*, Nanjing 11+*qEMF3* and IR64 were used as the donor and recurrent parent, respectively (Supplementary Figure S1B). A foreground screening of the BC_1_F_1_, BC_2_F_1_, and BC_3_F_1_ populations was conducted with the flanking SSR markers RM 14360, RM 14374, and RM 14394 to confirm the *qEMF3* genotype (Fig. 2A). A total of 82 SSR markers showing polymorphism between IR64 and Nanjing 11 were used for a background survey of 98 BC_3_F_1_ plants. An individual plant with a clear IR64 background (except in the *qEMF3* region) was selected and self-pollinated to proceed to the BC_3_F_2_ generation. BC_3_F_2_ plants with the EMF20 alleles at flanking markers for *qEMF3* were selected and advanced to BC_3_F_3_ to obtain seeds of NILs for *qEMF3* in the IR64 genetic background (IR64+*qEMF3*). The flowering patterns of IR64 and IR64+*qEMF3* were evaluated under flooded-field conditions in the wet season of 2013 (for 4 days in the middle of September) and in the dry season of 2014 (for 3 days in the middle of February) at the International Rice Research Institute (IRRI), Los Baños, the Philippines (14° 11’N, 121° 15’E). Plants were grown as described by Fujita *et al.* (2013). Spikelets that opened during the experiment were marked using fine-tipped pens every 30min, from 07.00 until 13.30. Four panicles from four individual plants were used for each genotype per day. The time from dawn to 50% of the FOT (T50) was calculated as described below to compare FOTs between IR64 and IR64+*qEMF3*.

### Greenhouse experiment with elevated day temperatures

Nanjing 11 and Nanjing 11+*qEMF3* were used to observe flowering patterns and to test sterility by using the method of Ishimaru *et al.* (2010). The experiment was conducted in 2010 in a glasshouse with clear-paned windows at NICS. Air temperature was manually increased by 2.0–3.5°C per hour after 08.00h. Maximum temperature reached ~40°C around noon. Air temperature was monitored every 15min with dataloggers (SK-L200TH II, Sato Keiryoki Mfg., Tokyo, Japan). Opened spikelets were marked with fine-tipped pens every hour. All pots were returned to the open-windowed greenhouse after marking. Sterile spikelets were manually counted at maturity.

### Heat tolerance test in an environmentally controlled growth chamber

Nanjing 11, Nanjing 11+*qEMF3*, IR64, and IR64+*qEMF3* were grown in 10-l pots. Plants were moved into walk-in growth chambers (3.3×3.2×2.7 m, or 10.6 m^2^ area), which provided a photosynthetic photon flux density of 1000 µmol m^–2^ s^–1^ at panicle height. For quantifying heat tolerance at flowering, heading panicles were exposed for 6h to 30°C (control) or 38°C (high temperature) at 60% relative humidity. The temperature treatment was imposed between 07.00 and 13.00h for Nanjing 11+*qEMF3* and IR64+*qEMF3*, and between 09.00 and 15.00h for Nanjing 11 and IR64 because of the difference in FOT between the recurrent parents and the EMF lines. Spikelets that flowered in the growth chamber during temperature treatment were marked, and after the end of the treatment period, all pots were returned to the non-stress greenhouse conditions. No spikelet flowering was observed before and after the temperature treatment. Sterility of the marked spikelets was estimated by manually counting the filled and unfilled spikelets.

### Response of IR64 and IR64+*qEMF3* to elevated high temperatures

Twelve seeds were regularly and circumferentially sown in 10-l pots using the method of Satake (1972). Plants were trimmed to the main stem once a week and grown in a greenhouse until the flowering stage. The flowering pattern was investigated in the walk-in chamber under three settings of temperature regime: (1) 25°C at 06.00, linearly increasing to 40°C by 14.00, then gradually decreasing to 25°C by 19.00; (2) 30 °C at 06.00, linearly increasing to 40°C by 14.00h, then gradually decreasing to 30°C by 19.00h; and (3), as (1) except that the temperature maximum (40°C) was reached at 12.00h. The lights were on from 06.00h until 19.00h; relative humidity was constant (60%) during the light period. Two or three panicles with the same heading date were selected per pot. Opened spikelets were marked manually every 30min after 06.00h. Eight panicles from three or four pots were used per genotype per day. Actual values of temperature and humidity were monitored every 15min with data loggers. After each temperature treatment, pots were returned to the greenhouse. To acclimate the plants to chamber conditions before the experiments, new pots were moved from the walk-in chamber maintained at 25°C or 30°C to the walk-in chamber used for this experiment at 19.00h. No flowering was observed before 06.00h. The experiment was repeated for 3 days for each temperature regime.

### Plant material and determination of FOT of popular local cultivars

The flowering patterns of cultivars listed in Table 3 were investigated in a naturally illuminated greenhouse at IRRI. We selected 23 popular cultivars from Southeast Asia (five cultivars), South Asia (five cultivars), Africa (eight cultivars), and Latin America (five cultivars) on the basis of nomination by the breeders in the CGIAR centres (IRRI, AfricaRice, and CIAT; International Centre for Tropical Agriculture) and at Tamil Nadu Agricultural University (Southern India). Nanjing 11, Nanjing 11+*qEMF3*, and CG14 (an EMF cultivar of *O. glaberrima*; Jagadish *et al.*, 2008) were also used. Nanjing 11+*qEMF3* was used as a control for the EMF trait. Plants were grown during the dry seasons of 2012 and 2013 at IRRI. Two 3-week-old seedlings were transplanted into a 10-l pot filled with 7.0kg of clay loam soil with a basal dressing of N (0.42g), P_2_O_5_ (0.42g), and K_2_O (0.42g) in each pot. Additional N (0.63g) was applied as top-dressing at 2 and 4 weeks after transplanting. Plants were grown under flooded conditions throughout the experiments. Standard plant protection measures at IRRI were followed; plants did not have any pest or disease damage. Experiments were conducted only on sunny days. More than three panicles (one panicle per plant) were used per genotype per day. Opened spikelets were marked every 30min from 07.30 (before the start of flowering) to 14.00 (until the end of flowering) using fine-tipped pens. Heading dates for different cultivars ranged from 22 February to 3 May. Nanjing 11+*qEMF3* was sown at weekly intervals from December until March to synchronize its heading date with that of each cultivar. Air temperature and relative humidity were monitored every 30min from 07.30 to 14.00 with data loggers (Supplementary Table S1). The experiment was repeated for at least three consecutive days for each genotype.

### Calculation of the beginning, peak, and end of FOT

FOT was included in the ‘time (hour) after dawn’ criterion because of the wide range of genotype heading dates and difference in natural day-length between wet and dry seasons. The exact time of dawn was calculated using the Koyomi Station software on the website of the Ephemeris Computation Office, Public Relations Centre, National Astronomical Observatory of Japan (http://eco.mtk.nao.ac.jp/koyomi/index.html.en). The time of dawn changed from 06.49 to 06.05 during the experimental period (from 22 February to 3 May). The flowering pattern was presented as percentage of opened spikelets on each day (*y*-axis) plotted against time after dawn (*x-*axis) (Supplementary Figure S2A). Curves were fitted by probit analysis using the R program (ver. 2.15.1) based on the cumulative percentage of opened spikelets (Supplementary Figure S2B and S2C). The times from dawn to 10% of the FOT, to 50% of the FOT, and to 90% of the FOT were calculated and defined as T10, T50, and T90, respectively. T10, T50, and T90 were also calculated using the R program. 

### Statistical analyses

The differences of means were analysed using the *t*-test or Tukey–Kramer test implemented in the R package (ver. 2.15.1).

## Results

### Identification of QTLs for EMF

**Fig. 1.**
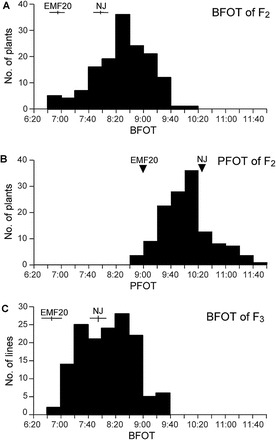
Frequency distribution of (A) BFOT and (B) PFOT for the F_2_ population derived from a cross between EMF20 and Nanjing 11. (C) Frequency distribution of the averaged BFOT in F_3_ lines (*n* = 10/line). The mean BFOT ± SD (*n* = 3) for EMF20 and Nanjing 11 (NJ) are shown in (A) and (C). The PFOTs for EMF20 and Nanjing 11 are shown with arrowheads in (B).

In 2007, the mean BFOT was 07.00 in EMF20 and 08.10 in Nanjing 11. The BFOT and PFOT of the 146 F_2_ plants from a cross between EMF20 and Nanjing 11 showed a normal frequency distribution (Fig. 1A, B). By using composite interval mapping analysis with 154 SSR markers and the 2007 data, we identified a significant QTL for BFOT (LOD score of 3.3) on chromosome 8, which explained 12.3% of the phenotypic variation (Table 1). For PFOT, two QTLs were identified on chromosomes 3 (LOD score of 3.8) and 8 (LOD score of 5.2), which explained 12.9 and 13.0% of the phenotypic variation, respectively (Table 1). In 2008, BFOT of the 146 F_3_ lines derived from F_2_ individuals was distributed normally (Fig. 1C). QTLs for BFOT were identified on chromosome 3 (LOD score of 8.4; 19.7% of phenotypic variation explained), chromosome 6 (LOD score of 2.5; 4.8% of phenotypic variation), and chromosome 8 (LOD score of 5.6; 12.9% of phenotypic variation) (Table 1). In both the F_2_ and F_3_ populations, the EMF20 alleles of QTLs on chromosomes 3 and 8 advanced FOT to early in the morning; these two QTLs were designated as *qEMF3* and *qEMF8* (because of the QTL for *Early-Morning Flowering*), respectively.

**Table 1.** QTLs for the beginning and peak of FOT

Year	Trait	Chromosome	Nearest- Marker	LOD score	AE^a^	VE^b^
2007	BFOT	3	RM14407	2.4	–16.0	7.7
		8	RM22459	3.3	–17.6	12.3
	PFOT	3	RM14407	3.8	–19.0	12.9
		8	RM22459	5.2	–7.8	13.0
2008	BFOT	3	RM14407	8.4	–23.7	19.7
		6	RM19715	2.5	4.3	4.8
		8	RM22459	5.6	–17.7	12.9

^a^ Additive effect of the EMF20 allele.

^b^ Variance explained (%).

### FOT of NILs carrying *qEMF3*

**Fig. 2.**
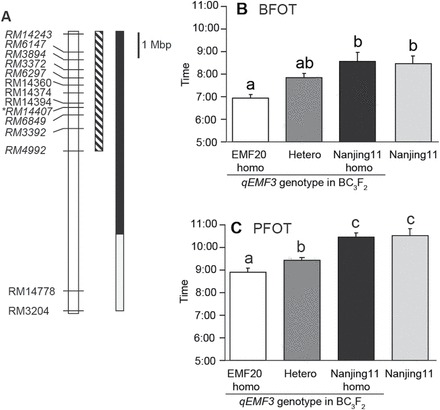
(A) Graphical genotype of the short arm of chromosome 3 used for the selection of lines for segregation analysis. Markers used for segregation analysis for FOT in (B) and (C) are italicized. The box with slanted lines indicates the region which showed LOD scores greater than the threshold. *RM14407 is the nearest marker to *qEMF3* (see Table 1). (B, C) Segregation analysis for FOT among selected genotypes of BC_3_F_2_ plants. Values shown are mean ± SE (*n* = 10). Values labelled with the same letters do not differ significantly. The Tukey–Kramer test (*P* < 0.05) was used for multiple comparisons between genotypes.

Segregation analysis for BFOT and PFOT was performed by using BC_3_F_2_ plants that were either EMF20 homozygous, Nanjing 11 homozygous, or heterozygous in the *qEMF3* region (Fig. 2A). The BFOT of EMF20-homozygous plants (06.54) was significantly earlier than that of Nanjing 11 (08.23; i.e. an 89-min difference), and also earlier than that of heterozygous plants (07.47), although the latter difference did not reach statistical significance (Fig. 2B). The BFOTs of Nanjing 11-homozygous plants (08.30) and Nanjing 11 were similar. The PFOTs of the EMF20-homozygous (08.50), heterozygous (09.21), Nanjing 11-homozygous (10.20), and Nanjing 11 (10.24) followed a pattern similar to that of BFOT (Fig. 2C). The results confirmed that a single QTL for EMF, *qEMF3*, significantly advanced the FOT of Nanjing 11 by ~1.5h.

### Nanjing 11 vs Nanjing 11+*qEMF3*: elevated temperature experiment in a greenhouse and heat tolerance test

**Fig. 3.**
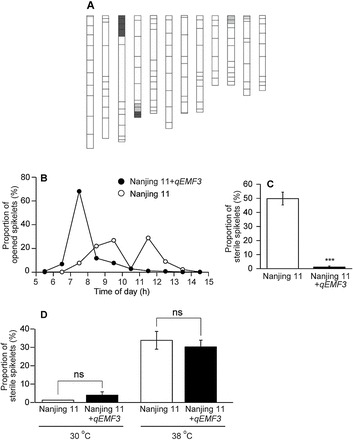
(A) Graphical genotype of Nanjing 11+*qEMF3* for the whole genome region. White bars, Nanjing 11, homozygous; grey bars, Nanjing 11, heterozygous; black bars, EMF20, homozygous. (B) and (C) Impact of *qEMF3* on flowering pattern and spikelet sterility in the Nanjing 11 genetic background. (B) Hourly changes in the percentage of opened spikelets on a single day. (C) Percentage of sterile spikelets at maturity under elevated temperature conditions in a greenhouse test (Table 2). 144 spikelets from five panicles were used for Nanjing 11 and 221 spikelets from seven panicles for Nanjing 11+*qEMF3*. Error bars indicate SE; ***, significant at the 0.1% level (*t*-test). (D) Percentage of sterile spikelets after temperature treatment at flowering in a growth chamber test. Under control conditions (30°C), 107 spikelets (Nanjing 11) and 131 spikelets (Nanjing 11+*qEMF3*) from five panicles were used. Under high-temperature conditions (38°C), 264 spikelets (Nanjing 11) and 238 spikelets (Nanjing 11+*qEMF3*) from eight panicles were used. Bars indicate SE; ns, not significant at the 5% level (*t*-test).

To prove the effectiveness of *qEMF3* in escaping heat stress at flowering, the NIL developed (Fig. 3A) was subjected to the elevated high temperature from early morning to noon in the greenhouse. In this experiment, air temperature steadily increased from ~28.0°C to ~34.0°C by 10.00, then exceeded 35.0°C, which is generally the threshold for induction of spikelet sterility (Satake and Yoshida, 1978; Matsui *et al.*, 1997), and reached 38.0–40.0°C at 11.00–15.00 (Table 2). The relative humidity steadily decreased (from >80.0% at 06.00 to ~40.0% by 15.00; Table 2). Nanjing 11+*qEMF3* clearly showed peak flowering at 07.00–08.00 and completed flowering by 11.00, whereas Nanjing 11 started flowering after 07.00, showed two peaks (09.00–10.00 and 11.00–12.00), and finished flowering by 14.00 (Fig. 3B). After 10.00, only 4.1% of spikelets in Nanjing 11+*qEMF3* flowered, whereas 43.1% of spikelets flowered in Nanjing 11. The percentage of sterile spikelets was significantly lower in Nanjing 11+*qEMF3* (1.2%) than in Nanjing 11 (49.8%) (Fig. 3C). In a chamber experiment for heat tolerance set constantly at 38°C for 6h on the day of flowering, spikelet sterility was similar between Nanjing 11 (33.9%) and Nanjing 11+*qEMF3* (30.3%) (Fig. 3D). Thus, Nanjing 11+*qEMF3* escaped heat-induced spikelet sterility by flowering earlier in the morning (i.e. at cooler temperatures).

**Table 2.** Time course of air temperature and relative humidity in the naturally illuminated greenhouse in 2010

	05.00–06.00	06.00–07.00	07.00–08.00	08.00–09.00	09.00–10.00	10.00–11.00	11.00–12.00	12.00–13.00	13.00–14.00	14.00–15.00
Temperature (^o^C)	27.7	28.0	29.2	32.6	34.3	37.1	39.0	37.6	38.8	40.8
Relative humidity (%)	82.9	81.4	77.8	68.4	65.7	57.2	49.2	51.3	48.4	42.1

Air temperature and relative humidity were logged every quarter hour and averaged hourly.

### IR64 vs IR64+*qEMF3*: flowering patterns at elevated high temperatures

**Fig. 4.**
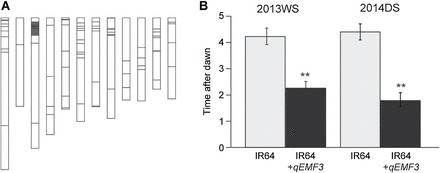
(A) Graphical genotype of IR64+*qEMF3,* a NIL for EMF in the IR64 genetic background. Chromosome segments from IR64 are shown in white; those from Nanjing 11+*qEMF3* are shown in black. (B) The mean T50 values ± SE for IR64 and IR64+*qEMF3* are shown for 4 days in the wet season (WS) of 2013 and 3 days in the dry season (DS) of 2014; plants were grown in the IRRI field.

**Fig. 5.**
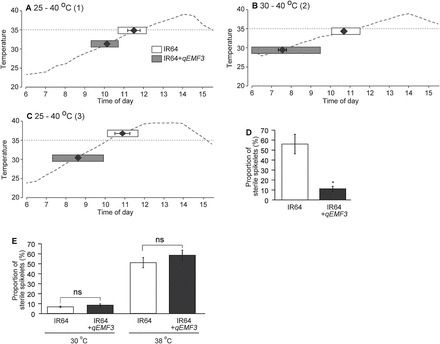
(A–C) Changes in FOT at elevated temperatures. (D) Spikelet sterility under the third temperature regime. (E) Heat tolerance of IR64 and IR64+*qEMF3*. (A) 25°C (actual 23.3°C at 06.00) to 40°C (actual 39.1°C at 14.00); (B) 30°C (actual 29.4°C at 06.00) to 40°C (actual 39.1°C at 14.00); (C) 25°C (actual 23.8°C at 06.00) to 40°C (actual 39.3°C at 12.00). Horizontal bars indicate the duration of flowering from T10 to T90. Closed circles in the horizontal bars indicate the mean T50 values ± SE. (D) In a total of 3 days of the experiment, 844 spikelets from 24 panicles were used for IR64 and 855 spikelets from 26 panicles were used for IR64+*qEMF3*. An asterisk indicates significant difference at the 5% level (*t-*test). (E) For the control treatment (30°C), 317 spikelets from six panicles were used for IR64 and 248 spikelets from six panicles were used for IR64+*qEMF3*. For the heat treatment (38°C), 388 spikelets from 10 panicles were used for IR64 and 326 spikelets from 11 panicles were used for IR64+*qEMF3*. ns, not significant at the 5% level (*t-*test).

We also transferred the EMF20 allele of *qEMF3* into IR64 through marker-assisted selection (Supplementary Figure S1B). The graphical genotype of the NIL developed (IR64+*qEMF3*) is shown in Fig. 4A. Under field conditions at IRRI, mean T50 of IR64+*qEMF3* was 1.9h in the wet season and 2.2h in the dry season (Fig. 4B), also confirming the effectiveness of *qEMF3* in the genetic background of IR64. These T50s were significantly earlier (by ~2h) than those of IR64. The IR64+*qEMF3* plants were further tested in the environmentally controlled chamber to understand the response of FOT to the elevated temperature. In the first temperature regime, T10 of IR64 and IR64+*qEMF3* was ~11.00 and ~09.30, respectively, T50 was ~11.30 and ~10.30, and T90 was ~12.30 and ~11.00 (Fig. 5A). Temperature at T90 was ~35.5°C (IR64) and ~33.0°C (IR64+*qEMF3*). In the second temperature regime, T10 of IR64 and IR64+*qEMF3* was ~10.00 and ~06.30, respectively, T50 was ~11.00 and ~07.30, and T90 was ~11.30 and ~09.30 (Fig. 5B). Temperature at T90 was ~35.5°C (IR64) and ~33.0°C (IR64+*qEMF3*). Spikelet opening started within 30min after light exposure in IR64+*qEMF3*. In the third temperature regime, which was the most severe among the three regimes, T10 of IR64 and IR64+*qEMF3* was ~10.30 and ~07.30, respectively, T50 was ~11.00 and ~09.00, and T90 was ~12.00 and ~10.00 (Fig. 5C). Temperature at T90 was ~38.5°C (IR64) and ~34.0°C (IR64+*qEMF3*). Spikelet sterility in the third temperature regime was significantly lower in IR64+*qEMF3* (11.2%) than in IR64 (55.8%) (Fig. 5D). Notably, the temperature at T90 for IR64+*qEMF3* was below the sterility threshold (35°C) in all three temperature regimes. In the heat tolerance test constantly at 38°C for 6h on the day of flowering, sterility was similar in IR64 (50.2%) and IR64+*qEMF3* (57.6%) (Fig. 5E).

### Comparison of FOT between Nanjing 11+*qEMF3* and popular local cultivars in the tropics and subtropics

**Fig. 6.**
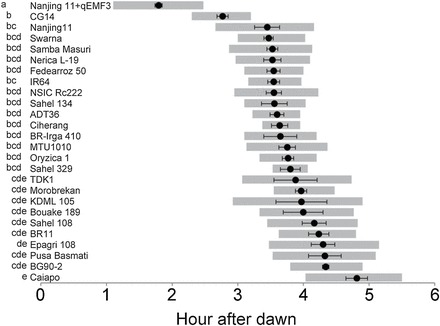
Comparison of time to 10% (T10), 50% (T50) and 90% (T90) after dawn in Nanjing 11+*qEMF3* and popular rice cultivars grown in the tropics and subtropics. Grey bars indicate time from T10 to T90 for each cultivar. T10, T50, and T90 calculations are shown in Supplementary Figure S2. The same letters on the left side of the cultivar names indicate no significant difference in T50. The Tukey–Kramer test (*P* < 0.05) was used for multiple comparisons between groups.

The mean T50 ranged from 3.4 to 4.8h after dawn (HAD) among popular cultivars (Table 3) in the dry season (Fig. 6). Swarna, an Indian cultivar, had the earliest T50 among the tropical and subtropical cultivars. Nanjing 11 and IR64 were placed in the early-T50 group. Caiapo, an upland cultivar from Latin America, had the latest T50 (3h later than Nanjing 11+*qEMF3*). The mean T50 of Nanjing 11+*qEMF3* was 1.8 HAD, which was significantly earlier than those of all tested cultivars (Fig. 6). The difference between T10 and T90 varied among the accessions: the difference was >1.5h for Nanjing 11, TDK1, KDML105, Epagri108, Pusa Basmati, and Caiapo, and was less for Ciherang and Sahel 329. For Nanjing 11+*qEMF3*, the mean T10 was 1.1 HAD and the mean T90 was 2.5 HAD; this T90 was earlier than T10 of all other cultivars. This result indicates that Nanjing 11+*qEMF3* had almost completed flowering prior to the beginning of FOT of the tested popular cultivars. The T50 of CG14 was 40min to 2h earlier than those of other cultivars but significantly later than that of Nanjing 11+*qEMF3*; T90 of Nanjing 11+*qEMF3* was similar to T10 of CG14 (Fig. 6).

**Table 3.** Cultivars and lines used in this study

Name of cultivar or line	Accession no.	Cultivar group	Area	Main countries of cultivation
Nanjing11+*qEMF3*	IRIS251-49436			
Nanjin11	IRIS251-49437	*Indica*-type	East asia	China
IR64	IRGC 66970	*Indica*-type	Southeast asia	Bhutan, Burkina Faso (FKR42), Cambodia, Ecuador (INIAP11), Gambia, India, Indonesia, Mauritania
NSIC Rc222	IRTP 24370	*Indica*-type	Southeast asia	The Philippines
Ciherang	IRTP 25143	*Indica*-type	Southeast asia	Indonesia
KDML 105	IRTP 6886	*Indica*-type	Southeast asia	Thailand
TDK1	IRIS 1-3354		Southeast asia	Laos
BR11	IRIS 109–4361		South asia	Bangladesh
MTU1010	IRTP 23154	*Indica*-type	South asia	India
Pusa Basmati	IRIS 10-90048		South asia	India
Swarna	IRTP 12715	*Indica*-type	South asia	India
ADT36	IRGC 64818	*Indica*-type	South asia	India
Sambha Mahsuri	IRTP 24472	*Indica*-type	South asia	India
BG90-2	IRGC 116958	*Indica*-type	Africa	Mali, Gambia, Ethiopia, Nigeria
Bouake 189	IRGC 78169	*Indica*-type	Africa	Ivory Coast, Guinea
Moroberekan	IRGC 12048	*Japonica*-type	Africa	Ivory Coast
Nerica L-19	IRIS 253-895327	*Indica*-type	Africa	Nigeria, Mali, Burkina Faso, Liberia, Sierra Leone, Cameroon, Togo
Sahel 108	IRIS 251–34260		Africa	Senegal, Mauritania
Sahel 134	WAB 15822	*Indica*-type	Africa	Senegal, Mauritania
Sahel 329	WAS 197-B-4-1-5	*Indica*-type	Africa	Senegal, Mauritania
BR-Irga 410	IRIS 294–6195	*Indica*-type	Latin America	Southern Brazil
Caiapo	IRIS 294–6354	*Indica*-type	Latin America	Northern Brazil
Epagri 108	IRIS 294–6315	*Indica*-type	Latin America	Southern Brazil, Venezuela
Fedearroz 50	IRIS 298–9831	Interspecific *indica* × *japonica*	Latin America	Colombia, Venezuela, Costa Rica, Panama
Oryzica 1	IRIS 294–5912	*Indica*-type	Latin America	Colombia
CG14	IRGC 96717	*O. glaberrima*	Africa	

The flowering patterns of CG14 were observed in 2013.

## Discussion

We attempted to develop NILs for EMF in *indica*-type genetic backgrounds through the detection of QTLs using a wild rice, *O. officinalis*, as a donor parent, aiming for genetic improvement of FOT to the cooler early morning. Heat-induced spikelet sterility is expected to aggravate rice yield losses in vulnerable temperate, subtropical, and tropical regions. We also investigated the FOTs of cultivars popular in tropical and subtropical rice-growing regions to identify the presence or absence of the EMF trait for the broader application of QTLs detected to rice breeding.

### *qEMF3* contributes to heat escape at flowering by advancing FOT to early morning

To date, there has been only one attempt to detect QTLs for EMF derived from *O. rufipogon* (AA genome; Thanh *et al.*, 2010); this study detected QTLs for BFOT on chromosomes 5 and 12, and QTLs for PFOT on chromosomes 4 and 5. However, the effects of these QTLs have not been functionally validated through the development of NILs, which makes it difficult to use these QTLs in breeding programmes. In our study, QTLs for BFOT and PFOT derived from *O. officinalis* were found on chromosomes 3 (*qEMF3*) and 8 (*qEMF8*) (Table 1); thus, we consider them to be different from those identified by Thanh *et al.* (2010). We confirmed the allelic effect of EMF20 on *qEMF3* for EMF in the BC_3_F_2_ population (Fig. 2B, C) and developed NILs for EMF in the genetic background of Nanjing 11 (Fig. 3A) and IR64 (Fig. 4A). Comparison of FOT between the recurrent parents and the NILs revealed that the EMF20 allele of *qEMF3* advanced FOT by 1.5–2.0h both in temperate Japan (Fig. 2B, C) and the tropical Philippines across different seasons (Fig. 4B), indicating that *qEMF3* advances the FOT under various environmental conditions.

Our previous study revealed that EMF20, an introgression line containing both *qEMF3* and *qEMF8*, avoids heat-induced spikelet sterility at flowering under an elevated temperature in the greenhouse (Ishimaru *et al.*, 2010). In this study, we found two QTLs for the EMF trait, *qEMF3* and *qEMF8* (Table 1). We tested the hypothesis that a single QTL, *qEMF3*, is effective in avoiding heat stress at flowering using Nanjing 11+*qEMF3* and IR64+*qEMF3*. By 10.00, when the temperature exceeded 35°C (Table 2), over 95% of spikelets had completed flowering in Nanjing 11+*qEMF3* but only 57% in Nanjing 11 (Fig. 3B). As a result, spikelet sterility was significantly reduced in Nanjing 11+*qEMF3* (Fig. 3C), although sterility was similar in Nanjing 11+*qEMF3* and Nanjing 11 in the heat tolerance test (Fig. 3D). This result supports our hypothesis that *qEMF3* is sufficient to advance the FOT of Nanjing 11, which allows this cultivar to escape heat stress during the daytime. Using an environmentally controlled chamber and three different temperature regimes, we confirmed that almost all spikelets of IR64+*qEMF3* flowered before the temperature reached 35°C, whereas the percentage of spikelets that opened at temperatures below 35°C varied in IR64 depending on the temperature regime (Fig. 5A–C). This difference in the flowering pattern significantly reduced spikelet sterility in IR64+*qEMF3* (Fig. 6D). The results clearly show that *qEMF3* has a similar effect on FOT in the IR64 background. Thus, *qEMF3* contributes to heat escape at flowering by advancing FOT. It is notable that *qEMF3* shifted its FOT drastically responding to the given temperature regimes (Fig. 5A–C). The NILs developed could be a novel material for revealing the interaction of genetic and environmental control of FOT.

### *qEMF3* has the potential to shift the FOT of cultivars popular in the tropics and subtropics to earlier in the morning

The vulnerability of the major rice-producing regions to heat-induced spikelet sterility at the flowering stage has been mapped both regionally (Wassmann *et al.*, 2009) and globally (Teixeira *et al.*, 2013). Although major local cultivars listed in Table 3 occupy a large proportion of rice cultivation areas in the tropics and subtropics, no assessment of their FOT traits has been conducted so far. Our investigation of the flowering patterns revealed that none of them had the EMF trait (Fig. 6). Among them, Nanjing 11 and IR64 were categorized in the early-FOT group (Fig. 6). The advancement in FOT due to *qEMF3* in the genetic background of Nanjing 11 (Fig. 2B, 2C; Fig. 7) and IR64 (Fig. 4B) shows that *qEMF3* has a significant impact even on the early-FOT cultivars.

Heat-induced spikelet sterility occurs even upon exposure of a flowering spikelet to a short-term heat stress (1h at ~37°C; Jagadish *et al.*, 2007). However, similar heat exposure 1h after flowering hardly affects fertility (Satake and Yoshida, 1978; Ishimaru *et al.*, 2010). Therefore, advancing FOT by 1h results in a significant difference in the proportion of sterile spikelets in rice under heat stress. Among the cultivars tested, Caiapo had the latest FOT, 3h later than Nanjing 11+*qEMF3* (Fig. 6). Two-thirds of the cultivars tested had their T50 2h later than Nanjing 11+*qEMF3* (Fig. 6), and these cultivars should be prioritized as targets for breeding programmes for EMF through the introgression of *qEMF3*. The fact that none of the popular cultivars tested, including CG14, which is known as an EMF cultivar (Jagadish *et al.*, 2008), had the same EMF trait as Nanjing 11+*qEMF3* shows a pressing need to transfer *qEMF3* to those cultivars by marker-assisted breeding. The allele of the EMF20 locus from *O. officinalis* has a potential to shift the FOT of these cultivars to a time earlier in the morning when the temperature is lower. Thus, the wild rice, *O. officinalis*, could provide an excellent genetic resource for the EMF trait to mitigate heat-induced spikelet sterility at flowering, which would help in breeding rice cultivars able to cope with future hotter climates.

## Supplementary Data

Supplementary Table 1. Meteorological data inside the greenhouse at IRRI in the 2012 and 2013 dry season.

Supplementary Figure S1. Breeding scheme for the development of NILs of rice (*O. sativa*) with the cultivars Nanjing 11 and IR64.

Supplementary Figure S2. An example of the T10, T50, and T90 calculation for three cultivars on a single day (22 February 2012).

## Funding

This study was supported by the Ministry of Foreign Affairs and the Ministry of Agriculture, Forestry, and Fisheries of Japan to T.I. and N.K., Federal Ministry for Economic Cooperation and Development, Germany to K.S.V.J., and the National Agricultural and Food Research Organization (NARO), Japan to T.I. and H.H.

## Supplementary Material

Supplementary Data

## Abbreviations:

BFOT: beginning of flower opening time
EMF: early-morning flowering
FOT: flower opening time
PFOT: peak of flower opening time.
