# Supplementary material for: qEMF3, a novel QTL for the early-morning flowering trait from wild rice, Oryza officinalis, to mitigate heat stress damage at flowering in rice, O. sativa
Source: J Exp Bot. 2014 Dec 22;66(5):1227–36. doi: 10.1093/jxb/eru474 (PMC4339588; doi:10.1093/jxb/eru474)
Supplement: Supplementary Data [file supp_66_5_1227__index.html]

 qEMF3, a novel QTL for the early-morning flowering trait from wild rice, Oryza officinalis, to mitigate heat stress damage at flowering in rice, O. sativa — qEMF3, a novel QTL for the early-morning flowering trait from wild rice, Oryza officinalis, to mitigate heat stress damage at flowering in rice, O. sativa — Supplementary Data 

# *qEMF3*, a novel QTL for the early-morning flowering trait from wild rice, *Oryza officinalis*, to mitigate heat stress damage at flowering in rice, *O. sativa*

## Supplementary Data

Data files

**Files in this Data Supplement:**

- Supplementary Data - Supplementary Data
