# Supplementary material for: qEMF3, a novel QTL for the early-morning flowering trait from wild rice, Oryza officinalis, to mitigate heat stress damage at flowering in rice, O. sativa
Source: J Exp Bot. 2014 Dec 22;66(5):1227–36. doi: 10.1093/jxb/eru474 (PMC4339588; doi:10.1093/jxb/eru474)
Supplement: Supplementary Data [file supp_eru474_jexbot136515_file001.pdf]

*qEMF3*, a novel QTL for early-morning flowering trait from wild rice, *Oryza officinalis*, to mitigate heat stress damage at flowering in rice (*Oryza sativa* L.)

Supplementary Table 1. Metrological data inside of greenhouse at IRRI in 2012 and 2013 dry season

| | 7:30 | 8:00 | 8:30 | 9:00 | 9:30 | 10:00 | 10:30 |
|---|---|---|---|---|---|---|---|
| Temperature (°C) | 28.7 ± 1.4 | 30.2 ± 1.7 | 31.5 ± 1.9 | 32.2 ± 1.9 | 33.1 ± 2.2 | 33.3 ± 2.6 | 33.6 ± 2.8 |
| Relative humidity (%) | 77.7 ± 6.1 | 72.3 ± 6.3 | 68.0 ± 5.6 | 64.8 ± 5.5 | 62.2 ± 7.1 | 60.6 ± 7.6 | 60.0 ± 8.9 |

Mean value ± S.E.

SupplementaryTable 2. continued

| | 11:00 | 11:30 | 12:00 | 12:30 | 13:00 | 13:30 | 14:00 |
|---|---|---|---|---|---|---|---|
| Temperature (°C) | 33.8 ± 2.7 | 34.2 ± 2.9 | 34.6 ± 2.8 | 34.7 ± 2.9 | 34.7 ± 3.2 | 34.3 ± 3.3 | 34.4 ± 3.0 |
| Relative humidity (%) | 58.9 ± 8.6 | 58.2 ± 9.3 | 56.4 ± 9.5 | 56.0 ± 9.2 | 56.6 ± 10.7 | 57.9 ± 10.8 | 57.9 ± 10.3 |

Values are mean ± SD of days for FOT investigation.

Hirabayashi et al. Fig. S1

*qEMF3*, a novel QTL for early-morning flowering trait from wild rice, *Oryza officinalis*, to mitigate heat stress damage at flowering in rice (*Oryza sativa* L.)

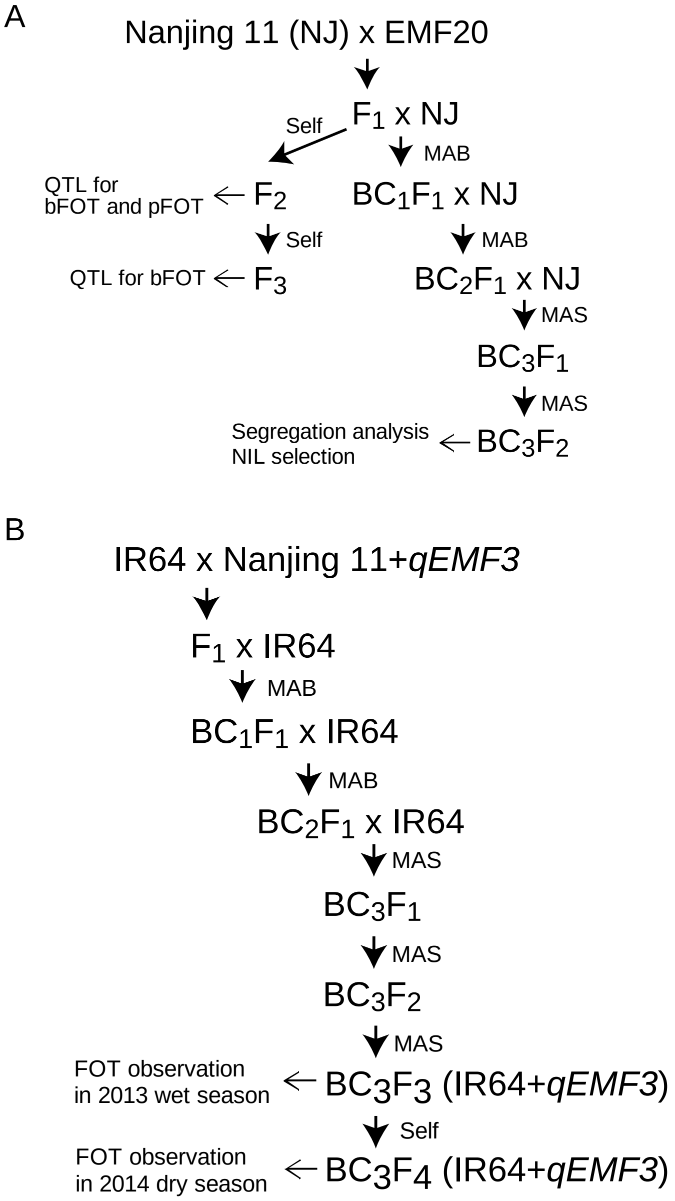


**Supplementary Figure S1.** Breeding scheme for the development of near-isogenic lines (NILs) of rice (*Oryza sativa* L.) with the cv. (A) Nanjing 11 and (B) IR64. The populations for quantitative trait locus (QTL) analysis for EMF locus are shown in A. MAB, marker-assisted backcrossing; MAS, marker-assisted selection.

Hirabayashi et al. Fig. S2

*qEMF3*, a novel QTL for early-morning flowering trait from wild rice, *Oryza officinalis*, to mitigate heat stress damage at flowering in rice (*Oryza sativa* L.)

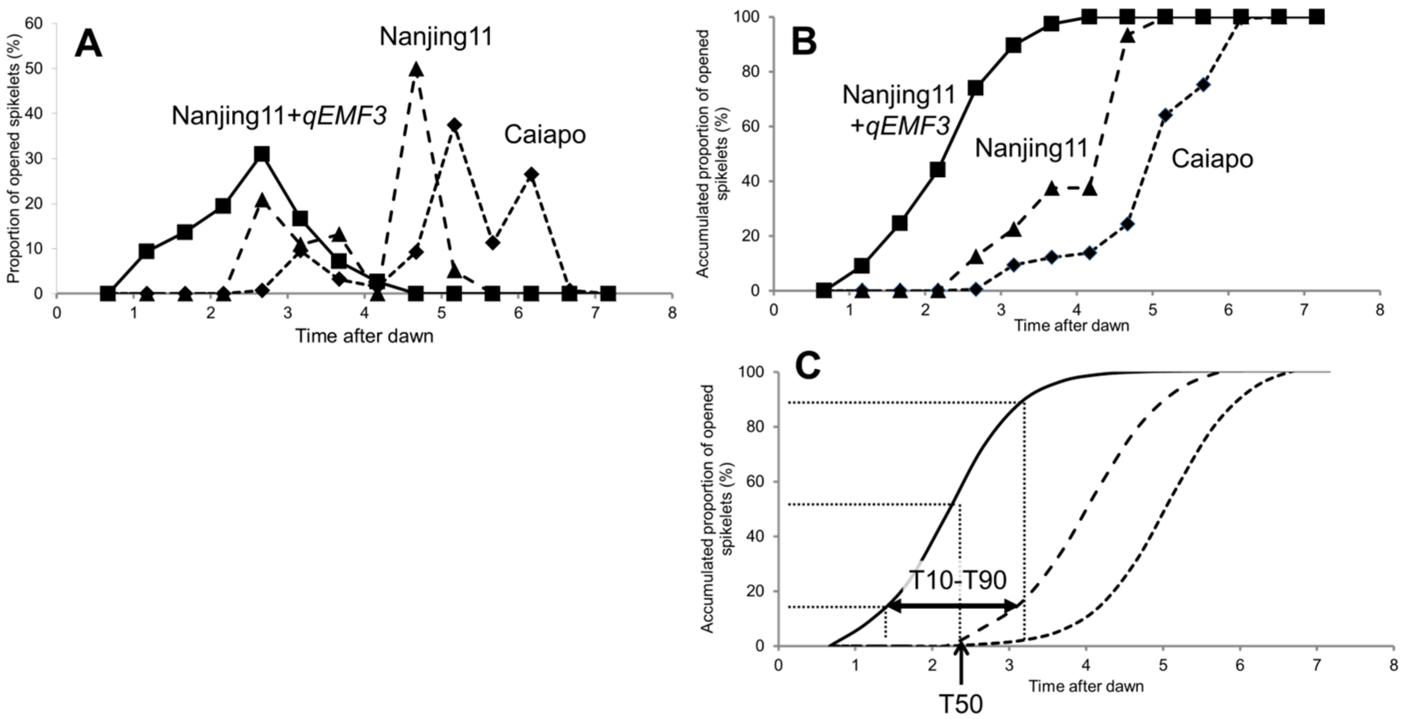


**Supplementary Figure S2.** An example of the T10, T50, and T90 calculation for three cultivars on a single day (22 February 2012). (A) Hourly changes in the percentage of opened spikelets. (B) Hourly changes in the accumulated percentage of opened spikelets (on the basis of S2A). (C) Fitting curves (on the basis of S2B).
